# Supplementary material for: Mendelian randomization analysis to identify potential drug targets for osteoarthritis
Source: PLoS One. 2025 Feb 11;20(2):e0316824. doi: 10.1371/journal.pone.0316824 (PMC11813149; doi:10.1371/journal.pone.0316824)
Supplement: S1 Table — (DOCX) [file pone.0316824.s001.docx]

**STROBE-MR checklist of recommended items to address in reports of Mendelian randomization studies**^1^ ^2^

| **Item No.** | **Section** | **Checklist item** | **Page No.** | **Relevant text from manuscript** |
| --- | --- | --- | --- | --- |
| 1 | **TITLE and ABSTRACT** | Indicate Mendelian randomization (MR) as the study’s design in the title and/or the abstract if that is a main purpose of the study | 1 | Mendelian randomisation analysis to identify potential drug targets for osteoarthritis |
|  | **INTRODUCTION** |  |  |  |
| 2 | **Background** | Explain the scientific background and rationale for the reported study. What is the exposure? Is a potential causal relationship between exposure and outcome plausible? Justify why MR is a helpful method to address the study question | 2 | In addition, Mendelian randomisation (MR) analysis, which uses single nucleotide polymorphisms (SNPs) identified by global genome analysis as instrumental variables (IVs) to explore the causal relationship between exposure and outcome, is considered to be an effective method for assessing causality, where exposure refers to some environmental or biological factor of interest in the study. MR analysis, which utilizes genotypes determined at the time of conception, effectively reduces the influence of confounding factors and thus clearly reveals causal effects. Compared to traditional observational studies, MR analysis can provide more unbiased estimates and has been widely used to identify drug targets and repurpose existing drugs. |
| 3 | **Objectives** | State specific objectives clearly, including pre-specified causal hypotheses (if any). State that MR is a method that, under specific assumptions, intends to estimate causal effects | 1 | we used Mendelian randomization analysis to identify circulating proteins that are causally associated with OA-related traits, providing important insights into potential drug targets for OA. |
|  | **METHODS** |  |  |  |
| 4 | **Study design and data sources** | Present key elements of the study design early in the article. Consider including a table listing sources of data for all phases of the study. For each data source contributing to the analysis, describe the following: | 2 | Osteoarthritis (OA) is the most common joint disease characterized by joint pain, swelling and dysfunction, which can lead to disability in severe cases. The disease primarily affects the knee, followed by the lumbar spine, cervical spine, hands, ankles and hips. The prevalence of OA is increasing every year as the population ages and the incidence of sports injuries increases, and the age of onset tends to be younger. However, the pathogenesis of OA has not been fully elucidated and no drugs have been successfully developed to slow its progression. Therefore, exploring the underlying mechanisms and drug targets of OA is of great value.Proteins are arguably the ultimate players in all life processes in disease and health. The human plasma proteome consists of proteins that are secreted or flow into the circulatory system, where they carry out their functions or mediate cross-tissue communication. Dysregulation of the human plasma proteome is a common symptom of a wide range of diseases, as is the case with OA. Given the importance of circulating proteins, they are an attractive resource for finding drug targets for OA. At the same time, plasma samples are easier to collect and less invasive than other tissues, and data on genetic variation in plasma proteins are richer and more readily available, making them an important source for identifying molecular markers of disease in large cohorts. |
|  | a) | Setting: Describe the study design and the underlying population, if possible. Describe the setting, locations, and relevant dates, including periods of recruitment, exposure, follow-up, and data collection, when available. | 3 | Figure1 B |
|  | b) | Participants: Give the eligibility criteria, and the sources and methods of selection of participants. Report the sample size, and whether any power or sample size calculations were carried out prior to the main analysis | 4 | Ferkingstad et al.'s study measured the genotype data for 4,907 plasma protein quantitative trait loci (pQTL) in 35,559 Icelandic individuals, which currently represents a more comprehensive analysis of plasma proteins. In addition, plasma pQTL data obtained from a recent study by Zheng et al were used for external validation.The genetic data for OA was sourced from a large-scale meta-analysis of GWAS in the UK Biobank (UKB), including five different sites of OA (knee OA, hip OA, spine OA, finger OA, and thumb OA) |
|  | c) | Describe measurement, quality control and selection of genetic variants | 4 | SNPs that are closely related to circulating proteins were selected in this study, with a threshold value of P < 5 × 10-8, in order to satisfy the correlation assumption.Subsequently, to address the issue of chain imbalance, the conditions of r2 < 0.001 and a window size of 10,000 kb were employed to ensure the independence of SNPs. Subsequently, palindromic sequences were removed by applying the harmonise_data() function, thereby ensuring greater reliability and accuracy of the genetic variants. Subsequently, the strong correlation of SNPs was assessed using the formula F = β2/SE2, where β represents the allele effect value and SE represents the standard error. The threshold for exclusion of weak instrumental variables was set at F<10. The same thresholds were applied to screen for significant SNPs in disease in inverse MR analysis. |
|  | d) | For each exposure, outcome, and other relevant variables, describe methods of assessment and diagnostic criteria for diseases | 3 | The utilisation of SNPs as IVs in MR analyses to assess causal associations between exposures (e.g., circulating proteins) and outcomes (e.g., knee OA and hip OA) necessitates the fulfilment of three fundamental assumptions（Figure1A）. |
|  | e) | Provide details of ethics committee approval and participant informed consent, if relevant |  |  |
| 5 | **Assumptions** | Explicitly state the three core IV assumptions for the main analysis (relevance, independence and exclusion restriction) as well assumptions for any additional or sensitivity analysis | 3 | Primarily, IVs must be demonstrably associated with exposure; secondly, IVs must not be associated with any confounding factors; and finally, IVs must influence outcomes exclusively through exposure |
| 6 | **Statistical methods: main analysis** | Describe statistical methods and statistics used |  |  |
|  | a) | Describe how quantitative variables were handled in the analyses (i.e., scale, units, model) |  | No applicable |
|  | b) | Describe how genetic variants were handled in the analyses and, if applicable, how their weights were selected | 4 | SNPs that are closely related to circulating proteins were selected in this study, with a threshold value of P < 5 × 10-8, in order to satisfy the correlation assumption.Subsequently, to address the issue of chain imbalance, the conditions of r2 < 0.001 and a window size of 10,000 kb were employed to ensure the independence of SNPs. |
|  | c) | Describe the MR estimator (e.g. two-stage least squares, Wald ratio) and related statistics. Detail the included covariates and, in case of two-sample MR, whether the same covariate set was used for adjustment in the two samples | 4 | Subsequently, palindromic sequences were removed by applying the harmonise_data() function, thereby ensuring greater reliability and accuracy of the genetic variants. Subsequently, the strong correlation of SNPs was assessed using the formula F = β2/SE2, where β represents the allele effect value and SE represents the standard error. The threshold for exclusion of weak instrumental variables was set at F<10. The same thresholds were applied to screen for significant SNPs in disease in inverse MR analysis. |
|  | d) | Explain how missing data were addressed |  | No missing data |
|  | e) | If applicable, indicate how multiple testing was addressed | 5 | In the preliminary analysis, Bonferroni correction was used for multiple testing adjustment, and a threshold pvalue (P < 0.05/number of proteins) was applied for result selection and further analysis. |
| 7 | **Assessment of assumptions** | Describe any methods or prior knowledge used to assess the assumptions or justify their validity |  | No applicable |
| 8 | **Sensitivity analyses and additional analyses** | Describe any sensitivity analyses or additional analyses performed (e.g. comparison of effect estimates from different approaches, independent replication, bias analytic techniques, validation of instruments, simulations) | 6 | Reverse causality test、Bayesian co localization analysis、Phenotype scanning、External validation |
| 9 | **Software and pre-registration** |  |  |  |
|  | a) | Name statistical software and package(s), including version and settings used | 4 | In this study, plasma pQTL and five specific outcomes were analysed by MR in R4.3.0 using the "TwoSampleMR" toolkit (https://github.com/MRCIEU/TwoSampleMR). |
|  | b) | State whether the study protocol and details were pre-registered (as well as when and where) |  | This study was not pre-registered. The main reason for this is that the study used a publicly available secondary dataset and the study design and analytical methods have been widely discussed and applied in the existing literature. Therefore, it is considered that pre-registration will not have a significant impact on the implementation and results of this study. |
|  | **RESULTS** |  |  |  |
| 10 | **Descriptive data** |  |  |  |
|  | a) | Report the numbers of individuals at each stage of included studies and reasons for exclusion. Consider use of a flow diagram |  | No applicable |
|  | b) | Report summary statistics for phenotypic exposure(s), outcome(s), and other relevant variables (e.g. means, SDs, proportions) | 6 | After selecting for genome-wide independence (r2 < 0.001, window size= 10000 kb) and P < 5 × 10-5, 1858 significant SNPs for 1553 proteins were screened; in the inverse MR analyses, the significant SNPs for the five OA's ranged from 1-38, the F-values of the significant SNPs for the proteins ranged from 36. 2 to 1479.6, and the significant SNPs for the OA had the smallest F-value of 29.7, all of which were greater than 10, indicating the lowest probability of weak instrumental bias, and the detailed information of each SNP was stored in (Table S1). |
|  | c) | If the data sources include meta-analyses of previous studies, provide the assessments of heterogeneity across these studies |  | No applicable |
|  | d) | For two-sample MR:  i.  Provide justification of the similarity of the genetic variant-exposure associations between the exposure and outcome samples  ii.  Provide information on the number of individuals who overlap between the exposure and outcome studies | 4 | Ferkingstad et al.'s study measured the genotype data for 4,907 plasma protein quantitative trait loci (pQTL) in 35,559 Icelandic individuals;The genetic data for OA was sourced from a large-scale meta-analysis of GWAS in the UK |
| 11 | **Main results** |  |  |  |
|  | a) | Report the associations between genetic variant and exposure, and between genetic variant and outcome, preferably on an interpretable scale |  | No applicable |
|  | b) | Report MR estimates of the relationship between exposure and outcome, and the measures of uncertainty from the MR analysis, on an interpretable scale, such as odds ratio or relative risk per SD difference | 6 | According to the Bonferroni-corrected threshold (P < 0.05/1553 = 3.22×10-5), the MR analysis revealed a total of 10 proteins (Table 2 and Table S2).OR greater than 1 in Table 2 indicates that an increase in the respective protein is associated with an increased risk of OA, while OR less than 1 suggests that an increase in the protein is associated with a decreased risk of OA. |
|  | c) | If relevant, consider translating estimates of relative risk into absolute risk for a meaningful time period |  | No applicable |
|  | d) | Consider plots to visualize results (e.g. forest plot, scatterplot of associations between genetic variants and outcome versus between genetic variants and exposure) | 7 | Table 2 MR Results of Plasma Proteins Significantly Associated with OA after Bonferroni Correction. |
| 12 | **Assessment of assumptions** |  |  |  |
|  | a) | Report the assessment of the validity of the assumptions |  | No applicable |
|  | b) | Report any additional statistics (e.g., assessments of heterogeneity across genetic variants, such as *I^2^*, Q statistic or E-value) | 6 | After selecting for genome-wide independence (r2 < 0.001, window size= 10000 kb) and P < 5 × 10-5, 1858 significant SNPs for 1553 proteins were screened; in the inverse MR analyses, the significant SNPs for the five OA's ranged from 1-38, the F-values of the significant SNPs for the proteins ranged from 36. 2 to 1479.6, and the significant SNPs for the OA had the smallest F-value of 29.7, all of which were greater than 10, indicating the lowest probability of weak instrumental bias, and the detailed information of each SNP was stored in (Table S1). |
| 13 | **Sensitivity analyses and additional analyses** |  |  |  |
|  | a) | Report any sensitivity analyses to assess the robustness of the main results to violations of the assumptions | 7 | Table 3 summarizes the main results of the reverse causal analysis, Bayesian colocalization analysis, and phenotype scan. |
|  | b) | Report results from other sensitivity analyses or additional analyses | 8 | For external validation, we conducted analyses using different variation and significance strategies within the same dataset. |
|  | c) | Report any assessment of direction of causal relationship (e.g., bidirectional MR) | 7 | Firstly, in the reverse MR analysis, except for IL12B which yielded no results, the pvalue calculated from the five methods for other proteins were all greater than 0.05, indicating no causal relationship between OA and the respective screened proteins. Furthermore, the Steiger filtering provided further confirmation of our results. |
|  | d) | When relevant, report and compare with estimates from non-MR analyses |  | No applicable |
|  | e) | Consider additional plots to visualize results (e.g., leave-one-out analyses) | 8 | Table 3 Reverse causal relationship detection, Bayesian co-localization analysis, and phenotype scanning results of 10 potential pathogenic protein.Table 4 External validation results using new plasma pQTL data. |
|  | **DISCUSSION** |  |  |  |
| 14 | **Key results** | Summarize key results with reference to study objectives | 9 | We employed a comprehensive analytical approach, integrating MR and colocalization techniques, to assess proteins associated with the pathogenesis of OA[21]. The objective is to identify biomarkers that can be utilized for early diagnosis and risk assessment. We acknowledge that the "causal relationships" identified through MR may be influenced by various factors, including reverse causality, horizontal pleiotropy, or genetic confounding[11]. Therefore, to minimize bias, we conducted bidirectional MR analyses, and the proteins identified in the preliminary MR analysis did not exhibit reverse causality. This conclusion was further supported by the Steiger filtering method[22]. Additionally, to limit bias introduced by horizontal pleiotropy, we only utilized cis-pQTLs as instruments, as they directly act in the transcription or translation processes[23]. |
| 15 | **Limitations** | Discuss limitations of the study, taking into account the validity of the IV assumptions, other sources of potential bias, and imprecision. Discuss both direction and magnitude of any potential bias and any efforts to address them | 12 | First, we examined the effects of proteins using data from different studies, where varying measurement methodologies may lead to biased results.Second, our analysis was confined to populations of European descent, limiting the generalizability of our results to other ethnic groups. |
| 16 | **Interpretation** |  |  |  |
|  | a) | Meaning: Give a cautious overall interpretation of results in the context of their limitations and in comparison with other studies | 10 | Therefore, to minimize bias, we conducted bidirectional MR analyses, and the proteins identified in the preliminary MR analysis did not exhibit reverse causality. This conclusion was further supported by the Steiger filtering method. Additionally, to limit bias introduced by horizontal pleiotropy, we only utilized cis-pQTLs as instruments, as they directly act in the transcription or translation processes. |
|  | b) | Mechanism: Discuss underlying biological mechanisms that could drive a potential causal relationship between the investigated exposure and the outcome, and whether the gene-environment equivalence assumption is reasonable. Use causal language carefully, clarifying that IV estimates may provide causal effects only under certain assumptions | 10 | Further phenotype scans revealed that SNPs associated with HHIP, ITIH1, MGP, IL12B, and RGMB were correlated with other traits. |
|  | c) | Clinical relevance: Discuss whether the results have clinical or public policy relevance, and to what extent they inform effect sizes of possible interventions | 13 | Our study culminated in the identification of four strongly correlated causal proteins in five OA's for which drug prediction.These findings not only aid in more precise early diagnosis and risk assessment but also offer new potential drug targets. This paves the way for developing treatment strategies tailored to various types of osteoarthritis. |
| 17 | **Generalizability** | Discuss the generalizability of the study results (a) to other populations, (b) across other exposure periods/timings, and (c) across other levels of exposure | 12 | First, we examined the effects of proteins using data from different studies, where varying measurement methodologies may lead to biased results.Second, our analysis was confined to populations of European descent, limiting the generalizability of our results to other ethnic groups. |
|  | **OTHER INFORMATION** |  |  |  |
| 18 | **Funding** | Describe sources of funding and the role of funders in the present study and, if applicable, sources of funding for the databases and original study or studies on which the present study is based | 13 | This manuscript was supported by funding from grants National Natural Science Foundation of China U23A6009, U21A20353, 82172503, Natural Science Foundation of Shanxi Province 20210302123285, Key R&D Program Projects of Shanxi Province 202202040201012, Hainan Provincial Medical and Health Research Program 21A200349.Chengyang Lu and Li Gu conceived and designed the project; Yanan Xu collects and processes data；Shuai Chen is responsible for drug screening and molecular docking；Chengyang Lu was responsible for drafting the manuscript, with revisions made by both Chengyang Lu and Pengcui Li on important content; Xiaochun Wei and Xueqin Rong provided critical feedback on important content. All authors participated in the writing of the paper and received final approval for the submitted and published versions. |
| 19 | **Data and data sharing** | Provide the data used to perform all analyses or report where and how the data can be accessed, and reference these sources in the article. Provide the statistical code needed to reproduce the results in the article, or report whether the code is publicly accessible and if so, where | 13 | The plasma pQTL data used for preliminary MR analysis in this study can be obtained from the deCODE Genetics website (https://www.decode.com/). The plasma pQTL data used for external validation are available for free access on the MR Base platform (www.mrbase.org). The GWAS data for osteoarthritis can be freely accessed through The Genetics of Osteoarthritis consortium (https://www.geneticsosteoarthritis.com). |
| 20 | **Conflicts of Interest** | All authors should declare all potential conflicts of interest | 14 | The authors declare no competing interests. |

This checklist is copyrighted by the Equator Network under the Creative Commons Attribution 3.0 Unported (CC BY 3.0) license.

1. Skrivankova VW, Richmond RC, Woolf BAR, Yarmolinsky J, Davies NM, Swanson SA, et al. Strengthening the Reporting of Observational Studies in Epidemiology using Mendelian Randomization (STROBE-MR) Statement. JAMA. 2021;under review.

2. Skrivankova VW, Richmond RC, Woolf BAR, Davies NM, Swanson SA, VanderWeele TJ, et al. Strengthening the Reporting of Observational Studies in Epidemiology using Mendelian Randomisation (STROBE-MR): Explanation and Elaboration. BMJ. 2021;375:n2233.
