# Supplementary material for: Mendelian randomization analysis to identify potential drug targets for osteoarthritis
Source: PLoS One. 2025 Feb 11;20(2):e0316824. doi: 10.1371/journal.pone.0316824 (PMC11813149; doi:10.1371/journal.pone.0316824)
Supplement: Attachment — Submitted filename: Review_PLOSONE.pdf [file pone.0316824.s009.pdf]

Summary

The authors conduct two-sample summary-data Mendelian randomization (MR) analyses to investigate the causal relationship between 1553 circulating proteins (as the exposure) and 5 osteoarthritis (OA) related traits (as the outcome). The causal effect of each protein on each OA trait is estimated using the Wald ratio (with a single variant) or inverse-variance weighted (IVW) approach (with multiple variants) as the main analysis and validated through a series of sensitivity checks, including reverse causality analyses, co-localization tests and phenotype searching.

The research question is well-defined, and the estimation strategy is clearly outlined. The initial MR results are effectively validated and filtered. Following the statistical analyses, a detailed discussion of the underlying biological mechanisms and drug target implications are provided. Please see my comments below.

Comments

- In Section 2.2, p.3, line.95, the citations for the OA GWAS data (Citation 12 and 13) may not be accurate. The correct reference appears to be Boer et al. (2021). Additionally, the description stating "....a large-scale meta-analysis of GWAS in the UK Biobank" (p.3, lns. 93-94) can be misleading, as the UK Biobank is only one of the cohorts included in the meta-analysis (Boer et al., 2021).
- Is there sample overlap between the protein and OA GWAS? For example, one of the 9 cohorts in the OA meta-analysis is Icelandic (cohort name: deCODE) (Boer et al., 2021), while the protein GWAS also utilizes Icelandic samples. It would be important to verify whether these represent two different Icelandic cohorts. Additionally, since the OA GWAS includes individuals from the UK Biobank, do any of the five protein

GWAS studies (listed in Citation 11) used for external validation also involve UK Biobank participants? Given that two-sample MR generally requires non-overlapping samples for the exposure and outcome, it is essential to investigate any potential sample overlap.

- In Section 2.3, p.5, line.107, I wonder whether it is necessary to screen SNPs using the $F < 10$ criterion. For a SNP with an effect size β and standard error SE, the t-statistic is given by $t = \frac{|\beta|}{SE}$. In a two-sided t-test, if a SNP passes the threshold of $p < 5 \times 10^{-8}$, then it implies that $t > 5.45$. Since the F statistic is calculated as $F = t^2$, it follows that $F > 5.45^2 > 10$. In other words, if the SNP screening procedure using the $p < 5 \times 10^{-8}$ criterion is already applied, then all SNPs that pass this criterion would inherently have $F > 10$. Therefore, it might be worth double-checking why the F statistic is still needed for IV selection after applying the genome-wide significance criterion.

- In Section 4, p.9, lines 241-243, it states that "we only utilize cis-pQTLs as instruments." However, there is no related description in the IV selection section (Section 2.3), nor is there any information on how the cis-pQTLs are selected. For example, in Citation 11, the authors define cis-acting pQTLs within a 500-kb window on either side of the leading pQTL of the protein (Zheng et al., 2020). How is this selection process conducted in this study?

- In Citation 11, the authors filtered out SNPs associated with more than five proteins as they might be highly pleiotropic, see the Methods section in Zheng et al. (2020). I wonder if this study conducted a similar procedure. If not, it might be helpful to provide information on the number of associated proteins for each pQTL. For example, the histogram in Panel A of Supplementary Figure 6 in Zheng et al. (2020) illustrates this well.

- In Section 2.6, p.5, line.134, should "share a single variant" be revised to "share a single

causal variant"? Additionally, in line 135, it would be helpful to clarify what hypothesis 4 is.

- In Section 2.6, p.5, lines 135-136, it states, "In PPH4, we examine...the shared variant region." How was the variant region defined? There is no related description provided.

- In Section 2.7, p.5, lines 145-146, what is meant by "priority protein pQTLs"? Additionally, are the results of the LD analysis presented anywhere in the paper? If so, please provide a reference; if not, please include them.

- In Section 3.2, p.6, line.172, it states that the Bonferroni adjusted p-value threshold is 0.05/1553, as there are 1553 proteins involved in the analysis for each OA trait. However, Supplementary Table 2 presents fewer than 1553 proteins with estimates. What is the reason for this discrepancy? Is it because fewer than 1553 proteins are used for assessing causality for each OA trait? If so, should the Bonferroni p-value threshold be adjusted accordingly? For example, for knee OA, there are 1164 proteins used for causal estimation, should the p-value threshold adjusted to 0.05/1164?

- In Section 3.3, p.7, line.186, what is the reason for the absence of reverse MR results for IL12B?

- In Section 3.3, p.7, line.188, "causal relationship between OA and the respective screened proteins" should be revised to "causal relationship from OA to the respective screened proteins" to indicate it is the reverse causal diretcion.

- It would be helpful to include the full estimation results of the reverse MR using all five MR methods, as well as the complete results of the external validation, in the supplementary materials.

- Some references seem to be absent. For example, additional references could be included in the Introduction when defining OA (p. 2, lines 43-44), discussing the trend

in OA prevalence (p. 2, lines 46-47), mentioning the statement "Dysregulation of the human plasma proteome... as is the case with OA" (p. 2, lines 53-54), and defining MR (p. 2, lines 61-63). Additionally, what are the references for MR-IVW and the four MR methods used in the reverse causality analyses (MR-Egger, weighted median method, simple mode, and weighted mode)? Also, what are the references for Steiger filtering and the Bayesian co-localization method?

- **Clarification questions:**

  1. In Abstract, p.1, line.20, should "Causal associations between 4907 circulating proteins and five OA-related traits...." be revised to "....between 1553 circulating proteins....", as after the IV selection procedure, only 1553 out of 4907 proteins are selected for MR analyses.
  2. In Abstract, p.1, lines 23-24, the statement "....inverse MR analysis and Steiger filtering to ensure positive associations between circulating proteins and OA" does not seem accurate. Why is it stated "to ensure positive associations"? My understanding is that these analyses are primarily used to assess the direction of causality. A similar statement appears in Section 2.5, p.5, In.127. "....Steiger filtering as a dual test to ensure a positive association....".
  3. In Abstract, p.1, line.25, what does "cascading effects" refer to?
  4. In Section 2.3, p.4, line.103, what is meant by "chain imbalance"?
  5. In Section 2.3, p.4, line.105, is "harmonise_data()" a function from the R package "TwoSampleMR"? It would be helpful to provide full details.
  6. In Section 2.4, p.4, line.115, what does "heterogeneity analysis" refer to? Does it refer to the analysis using the four MR methods in addition to MR-IVW?
  7. In Section 2.5, p. 5, line.128, what is meant by "SNP mismatch"? Does it refer to SNPs that influence the outcome (and are therefore correlated with the exposure) being used as IVs for the exposure?

8. In Section 2.8, p. 5, line.149, what is meant by "as a new exogenous exposure"? Does this refer to using the new proteins from Citation 11 as exposures? If so, the term "exogenous" could be misleading, as all the exposures in this context are endogenous and therefore require IVs for causal inference.
9. In Section 3.3, p. 7, line 184, the title of the subsection mentions "five causal proteins." Why is it five?
10. In Section 3.3, p. 7, lines 202-203, what is meant by "associations with themselves"?
11. In Section 6, p.12, line.347, why are the four causal proteins described as "strongly correlated"? If this refers to these proteins having significant estimates, the use of "correlated" could be misleading, as the final conclusion is that the proteins are likely to exert causal effects, rather than simply exhibiting correlations.

- The sheet names in Supplementary Table S1 are not in English.

References

Boer, C. G., Hatzikotoulas, K., Southam, L., Stefánsdóttir, L., Zhang, Y., de Almeida, R. C., Wu, T. T., Zheng, J., Hartley, A., Teder-Laving, M., et al. (2021). Deciphering osteoarthritis genetics across 826,690 individuals from 9 populations. *Cell*, *184*(18), 4784–4818.

Zheng, J., Haberland, V., Baird, D., Walker, V., Haycock, P. C., Hurle, M. R., Gutteridge, A., Erola, P., Liu, Y., Luo, S., et al. (2020). Phenome-wide mendelian randomization mapping the influence of the plasma proteome on complex diseases. *Nature genetics*, *52*(10), 1122–1131.
