# Supplementary material for: Mendelian randomization analysis to identify potential drug targets for osteoarthritis
Source: PLoS One. 2025 Feb 11;20(2):e0316824. doi: 10.1371/journal.pone.0316824 (PMC11813149; doi:10.1371/journal.pone.0316824)
Supplement: Attachment — Submitted filename: Review_PLOSONE_revision_1.pdf [file pone.0316824.s013.pdf]

Summary

The authors have effectively addressed the majority of my concerns and questions, but I have a few additional comments regarding their responses, which are listed below.

Further Comments

1. **Original comment:** In Section 2.2, p.3, line.95, the citations for the OA GWAS data (Citation 12 and 13) may not be accurate. The correct reference appears to be Boer et al. (2021). Additionally, the description stating "....a large-scale meta-analysis of GWAS in the UK Biobank" (p.3, Ins. 93-94) can be misleading, as the UK Biobank is only one of the cohorts included in the meta-analysis (Boer et al., 2021).

   Authors' response: Thank you for your comment. We have corrected the citations for the OA GWAS data in Section 2.2. The original references (Citations 12 and 13) were not primary sources, and we have now replaced them with the correct reference to Boer et al. (2021). Additionally, We also recognize that our description of the GWAS meta-analysis was incomplete. The UK Biobank is only one of the nine cohorts included in the analysis, and we have revised the text accordingly in the manuscript. (lines 94-97).

   Further comment: In the corrected citation for the OA GWAS (i.e., Boer et al. (2021)), the authors indicate that their GWAS identified risk variants for 11 OA-related phenotypes (as mentioned in the Summary section and the Graphical Abstract of the paper). However, in the revised manuscript, it is stated that the study involved "826,690 individuals (including 177,517 OA patients) and covered 12 OA-related traits" (p.3, line 96). Could you clarify why 12 OA traits are mentioned here, rather than 11 as stated in the cited paper? Additionally, please revise Figure 1B to reflect the correct reference for the OA data: Under "Outcome: Osteoarthritis", it currently lists

"Tachmazidou et al. (2019)" instead of "Boer et al. (2021)". Also, in Figure 1B, under "Exposure: Proteins", it might be misleading to state "35,559 controls", as proteins are not case-control phenotypes. It may be more accurate to describe this as "35,559 participants".

2. **Original comment:** Is there sample overlap between the protein and OA GWAS? For example, one of the 9 cohorts in the OA meta-analysis is Icelandic (cohort name: deCODE) (Boer et al., 2021), while the protein GWAS also utilizes Icelandic samples. It would be important to verify whether these represent two different Icelandic cohorts. Additionally, since the OA GWAS includes individuals from the UK Biobank, do any of the five protein GWAS studies (listed in Citation 11) used for external validation also involve UK Biobank participants? Given that two-sample MR generally requires non-overlapping samples for the exposure and outcome, it is essential to investigate any potential sample overlap.

   Authors' response: Thank you for your thoughtful question. We fully acknowledge the concerns you raised regarding potential sample overlap. Indeed, two-sample MR assumes that the exposure and outcome come from independent samples, and any sample overlap can impact the accuracy of causal inference results.

   First, regarding the Icelandic cohort you mentioned, while it is true that the OA GWAS analysis includes the Icelandic deCODE cohort, based on our current data, the Icelandic samples in the protein GWAS and OA GWAS do not come from the same population. We will further verify this to ensure that the samples used in these studies are independent. In addition, your point about the UK Biobank samples is important. While some UK Biobank samples were indeed used in the protein GWAS, there is not a complete overlap with the OA GWAS samples. To ensure the robustness of our analysis, we plan to use LD score regression (LDSC) to assess any potential sample overlap.

   LDSC provides important insights through the genetic correlation coefficient (r_g) and

SNP heritability (h^2) estimates. We will focus on the following key points:

(a) Genetic correlation coefficient (r_g) and its p-value: If the r_g is small (typically $|r_g| \leq 0.2$) and the p-value is non-significant ($p > 0.05$), we can reasonably assume that there is no significant genetic overlap between the samples. A small r_g value suggests that the traits are genetically uncorrelated, helping to rule out the possibility of sample overlap.

(b) SNP heritability (h^2): If the h^2 estimates for each trait fall within a reasonable range (0.05–0.2) and show no significant discrepancies between them, this further supports the independence of the samples.

Our analysis shows that the genetic correlation coefficients between the four identified proteins and OA are $|r_g| \leq 0.2$, with non-significant p-values ($p > 0.05$). Therefore, we can reasonably conclude that the samples used in our study are independent, and the impact of sample overlap is minimal.

We will include these results in our final manuscript and appreciate your attention and thoughtful feedback on our research.

Further comment:

- In the protein GWAS paper by Ferkingstad et al. (2021), the Methods section states: "We collected plasma samples from 40,004 Icelanders through two main projects: the Icelandic Cancer Project (52% of participants; samples collected from 2001 to 2005) and various genetic programs at deCODE genetics, Reykjavík, Iceland (48%)." In the OA GWAS paper by Boer et al. (2021), the authors also indicate that one of the cohorts used for meta-analysis is deCODE (refer to the Key Resources table in the paper). Given this overlap, could you please provide solid evidence supporting your claim that "the Icelandic samples in the protein GWAS and OA GWAS do not come from the same population"?
- It is a valid approach to investigate potential sample overlap using LD score regres-

sion. However, it is unclear to me why you chose to report the genetic correlations between the proteins and OA traits and the heritability of the proteins. According to Bulik-Sullivan et al. (2015) which introduced the cross-trait LD score regression for estimating the genetic correlation between two phenotypes, the regression equation is as follows (see Equation 1 in the paper):

$$E[z_{1j}z_{2j}] = \frac{\sqrt{N_1 N_2}\rho_g}{M} l_j + \frac{\rho N_s}{\sqrt{N_1 N_2}}.$$

Here, N_s represents the size of the overlapping samples. If there is no sample overlap ($N_s = 0$), then the intercept of the LD score regression (the term $\frac{\rho N_s}{\sqrt{N_1 N_2}}$) will be zero. The genetic correlation is estimated from the slope of the regression (the term $\frac{\sqrt{N_1 N_2}\rho_g}{M}$), where ρ_g is the genetic covariance, from which the genetic correlation can be derived. As stated in Bulik-Sullivan et al. (2015), "sample overlap only affects the intercept from this regression (the term $\frac{\rho N_s}{\sqrt{N_1 N_2}}$) and not the slope, so the estimates of genetic correlation will not be biased by sample overlap." (see the Overview of Methods section of the paper). Therefore, to properly investigate sample overlap, you should report the intercept from the LD score regression and test whether it is significantly different from zero, rather than the genetic correlation or heritability. For additional evidence supporting the use of the intercept to test for sample overlap, see, e.g., Yengo et al. (2018) and Lee et al. (2018).

- Please revise the rationale in Section 2.8 (p.6, lines.170-178) and the results in Section 3.4 (p.9, lines.242-246) accordingly after you analyze and report the intercept instead. Additionally, please provide more details on how the LD score regression was conducted, e.g., clarify whether the analysis uses the main protein data or the external validation protein data and which R package/functions are used for the analysis rather than simply stating "All analyses were conducted

using RStudio."

3. **Original comment:** In Section 2.3, p.5, line.107, I wonder whether it is necessary to screen SNPs using the $F < 10$ criterion. For a SNP with an effect size β and standard error SE, the t-statistic is given by $t = \frac{|\beta|}{SE}$. In a two-sided t-test, if a SNP passes the threshold of $p < 5 \times 10^{-8}$, then it implies that $t > 5.45$. Since the F statistic is calculated as $F = t^2$, it follows that $F > 5.45^2 > 10$. In other words, if the SNP screening procedure using the $p < 5 \times 10^{-8}$ criterion is already applied, then all SNPs that pass this criterion would inherently have $F > 10$. Therefore, it might be worth double-checking why the F statistic is still needed for IV selection after applying the genome-wide significance criterion.

   Authors' response: Thank you for raising this point. While it is correct that SNPs passing the genome-wide significance threshold ($p < 5 \times 10^{-8}$) would inherently have an F-statistic greater than 10, we have included the F-statistic criterion for consistency with previous studies and as a safeguard to ensure instrument strength in all contexts. This additional check helps us to confirm robust instrument selection across different datasets and analysis scenarios. Therefore, we believe it is still valuable to explicitly apply this criterion.

   Further comment: If there are no specific reasons for including the $F > 10$ criterion in the IV selection process, I do not believe it is necessary to retain this step. To my knowledge, it is standard practice in MR studies to apply only the genome-wide significance threshold ($p < 5 \times 10^{-8}$) to ensure instrument strength, as the $F > 10$ criterion becomes redundant under this threshold. For example, see the instrument selection steps described in the Methods section of Zheng et al. (2020) (citation 20). I recommend removing the $F > 10$ step from the IV selection procedure (p.4, lines 110–112) to avoid redundancy and align with standard practices.

4. **Original comment:** In Section 3.2, p.6, line.172, it states that the Bonferroni ad-

justed p-value threshold is 0.05/1553, as there are 1553 proteins involved in the analysis for each OA trait. However, Supplementary Table 2 presents fewer than 1553 proteins with estimates. What is the reason for this discrepancy? Is it because fewer than 1553 proteins are used for assessing causality for each OA trait? If so, should the Bonferroni p-value threshold be adjusted accordingly? For example, for knee OA, there are 1164 proteins used for causal estimation, should the p-value threshold adjusted to 0.05/1164?

Authors' response: Thank you for your comments and suggestions. I would like to clarify the reason for the number of proteins in Supplementary Table 2 being less than 1553: The number of proteins in Supplementary Table 2 is less than 1553 because not all pQTLs have corresponding SNPs for each OA trait, which results in fewer available proteins for actual causal estimation than 1553. Regarding the Bonferroni adjusted p-value threshold, we determined a total of 1553 proteins as the targets for preliminary analysis during the study design phase. Therefore, the Bonferroni adjusted p-value threshold is calculated based on this total number, specifically 0.05/1553. The fundamental principle of Bonferroni correction is to reduce the overall significance level (e.g., 0.05) by dividing it by the number of comparisons (in this case, 1553) to effectively control the first-type error rate. This helps ensure that the probability of incorrectly rejecting the null hypothesis during multiple hypothesis testing is minimized. Although fewer proteins (e.g., 1164) are used in some analyses, the Bonferroni adjusted threshold remains applicable. This is because the adjustment is designed to account for all potential hypothesis tests in the preliminary analysis. Maintaining this initial p-value threshold helps ensure the conservativeness and consistency of statistical results, thereby reducing the false discovery rate associated with multiple comparisons. In summary, we will keep the Bonferroni adjusted p-value threshold at 0.05/1553 to ensure the scientific rigor and statistical reliability of the results.

Further comment: I would like to clarify that the Bonferroni adjusted p-value should

be based on the total number of (independent) tests performed, rather than the nominal number of exposure phenotypes. If you are treating each set of MR analyses corresponding to each OA trait separately (and ignore the possible correlations between the proteins), the Bonferroni adjusted p-value should be calculated as 0.05 divided by the actual number of proteins used as exposures for that specific OA trait. For example, for knee OA, it should be 0.05/1164 rather than 0.05/1553. Alternatively, if you intend to account for all hypothesis tests jointly by applying a universal Bonferroni corrected p-value, you may need to consider the total number of tests across all OA traits. For example, when testing pairwise causality between 100 exposure traits and 90 outcome traits, the universal Bonferroni p-value would be $0.05/(100 \times 90)$, resulting in a more conservative threshold. As a reference, it seems that Zheng et al. (2020) (citation 20) applied this type of Bonferroni correction, leading to a stringent threshold of $p < 3.5 \times 10^{-7}$. This threshold is calculated as $0.05/142,857$ where $142,857$ is the total number of (independent) tests between all protein exposures and all outcome traits. This is considerably stricter than 0.05/1002 where 1002 is the number of protein exposures involved in their MR analyses. For more details and complications with defining the number of independent tests, see the subsection "Estimating the causal effects of proteins on human phenotypes using MR" in the Methods section of their paper. I would not insist on revising the current threshold p-value of 0.05/1553, as applying the more lenient OA-specific p-value (e.g., 0.05/1164 for knee OA) does not affect the significance of the current causal relationships. However, this might be an important point to consider in future research.

5. **Original comment:** In Section 3.3, p.7, line.186, what is the reason for the absence of reverse MR results for IL12B?

   Authors' response: Thank you for your question. I would like to clarify the reason for the absence of reverse MR results for IL12B: In the reverse MR analysis, there is only one significant SNP for Spine OA. However, this significant SNP does not

have a corresponding SNP in the IL12B data, which prevents us from using the harmonise_data() function to merge the data. As a result, due to the lack of matching SNPs, we are unable to conduct causal analysis.

Further comment: Thank you for the clarification. It would be helpful to also include an explanation in the main text, stating that this is due to a lack of data (Section 3.3, p.8, lines 213–214).

6. **Original comment:** It would be helpful to include the full estimation results of the reverse MR using all five MR methods, as well as the complete results of the external validation, in the supplementary materials.

   Authors' response: Thank you for your suggestion. We will include the full results of the reverse MR analysis and external validation in the supplementary materials. The reverse MR results will be uploaded as Table S3, and the complete external validation results will be uploaded as Table S5. These supplementary materials will provide readers with more detailed estimates and validation data to further understand our findings.

   Further comment: Thank you for providing the results. In Table S3, Column A, the IDs of the results are not in consecutive order, please revise them. In Table S5, Sheet "OR-Knee OA", Row 3, Column R, there is a number "6.81199E-05". Please either remove it or clarify its meaning and what it refers to. Additionally, it would be helpful to include the Steiger filtering results in Table S3 as well (in a separate sheet) so that this table provides a comprehensive summary of all the reverse causality results.

7. **Original comment:**In Abstract, p.1, lines 23-24, the statement "....inverse MR analysis and Steiger filtering to ensure positive associations between circulating proteins and OA" does not seem accurate. Why is it stated "to ensure positive associations"? My understanding is that these analyses are primarily used to assess the direction of causality. A similar statement appears in Section 2.5, p.5, In.127. "....Steiger filtering

as a dual test to ensure a positive association....".

Authors' response: Thank you for the valuable feedback. Our wording was indeed inaccurate. It is correct that inverse MR analysis and Steiger filtering are used to assess causal relationships. What we intended to convey is that our goal is to ensure that the direction of the causal relationship is from proteins to OA, while there should not be a causal relationship in the reverse direction, i.e., from OA to proteins. Therefore, we conducted these two analyses. We will revise the text to reflect this clarification more accurately.(lines 23-24).

Further comment: Thank you for your explanation, which is clear to me. However, I find the phrasing in the manuscript of "to ensure the positive causal relationship between circulating proteins and OA" (line 24 and line 142) to be misleading. It seems to suggest that the sign of the causal effects of the proteins on OA are positive. To avoid confusion, it would be clearer to revise the phrasing to "to ensure the causal direction between circulating proteins and OA."

8. A few questions about the referencing:

    - In Section 2.4, p.4, lines 122-123, the reference (citation 25) for the statement "This increases statistical power....from multiple independent variants" does not appear to be correct. Could you please clarify which specific part of citation 25 supports this statement? If it does not, please provide a more appropriate reference.
    - In Section 2.5, please add references for the statements related to MR-IVW, e.g., "Under the assumption that....precise estimates." (lines 135-136); "It is also the most statistically powerful....horizontal pleiotropy is absent." (lines 136-138).
    - In Section 2.5, p.5, lines 142-143, the reference for Steiger filtering (citation 25) does not appear to be correct. A more appropriate reference would be Hemani et al. (2017).

- In Section 2.6, p.5, lines 147-150, the reference (citation 29) for Bayesian colocalization does not appear to be correct. Based on the R package used for the colocalization analysis, the appropriate reference for the method seems to be either Giambartolomei et al. (2014) or Wallace (2021). Please further verify this and update the manuscript with the correct reference.
- In Section 2.6, lines 156-157, could you please clarify which specific part of citation 30 supports the posterior probability threshold of 0.8?
- In Section 2.8, p.6, lines 171–172, the reference (citation 32) for LD score regression is not the original source of the method. The correct reference should be Bulik-Sullivan et al. (2015). Please update the citation accordingly.
- I would suggest carefully reviewing the references and citations throughout the manuscript to ensure their accuracy.

9. A few questions about the writing:

   - p.5, line 146, the title of Section 2.6 “Bayesian co localization analysis” should be revised to “Bayesian colocalization analysis”.
   - p.5, line 156, “We set the posterior probability threshold to greater than 0.8” should be revised to “We set the posterior probability threshold to be greater than 0.8”.
   - p.6, line 167, “‘as a new endogenous exposure” should be revised to “as new endogenous exposures”.
   - p.8, line 211, the title of Section 3.3 “Sensitivity analysis of the causal relationships between proteins and five OA” should be revised to “Sensitivity analysis of the causal relationships between proteins and five OA phenotypes”.
   - I would suggest carefully reviewing the writing throughout the manuscript.

References

Boer, C. G., Hatzikotoulas, K., Southam, L., Stefánsdóttir, L., Zhang, Y., de Almeida, R. C., Wu, T. T., Zheng, J., Hartley, A., Teder-Laving, M., et al. (2021). Deciphering osteoarthritis genetics across 826,690 individuals from 9 populations. *Cell*, *184*(18), 4784–4818.

Bulik-Sullivan, B., Finucane, H. K., Anttila, V., Gusev, A., Day, F. R., Loh, P.-R., Consortium, R., Consortium, P. G., for Anorexia Nervosa of the Wellcome Trust Case Control Consortium 3, G. C., Duncan, L., et al. (2015). An atlas of genetic correlations across human diseases and traits. *Nature genetics*, *47*(11), 1236–1241.

Ferkingstad, E., Sulem, P., Atlason, B. A., Sveinbjornsson, G., Magnusson, M. I., Styrmisdottir, E. L., Gunnarsdottir, K., Helgason, A., Oddsson, A., Halldorsson, B. V., et al. (2021). Large-scale integration of the plasma proteome with genetics and disease. *Nature genetics*, *53*(12), 1712–1721.

Giambartolomei, C., Vukcevic, D., Schadt, E. E., Franke, L., Hingorani, A. D., Wallace, C., & Plagnol, V. (2014). Bayesian test for colocalisation between pairs of genetic association studies using summary statistics. *PLoS genetics*, *10*(5), e1004383.

Hemani, G., Tilling, K., & Davey Smith, G. (2017). Orienting the causal relationship between imprecisely measured traits using gwas summary data. *PLoS genetics*, *13*(11), e1007081.

Lee, J. J., McGue, M., Iacono, W. G., & Chow, C. C. (2018). The accuracy of ld score regression as an estimator of confounding and genetic correlations in genome-wide association studies. *Genetic epidemiology*, *42*(8), 783–795.

Wallace, C. (2021). A more accurate method for colocalisation analysis allowing for multiple causal variants. *PLoS genetics*, *17*(9), e1009440.

Yengo, L., Yang, J., & Visscher, P. M. (2018). Expectation of the intercept from bivariate ld score regression in the presence of population stratification. *BioRxiv*, 310565.

Zheng, J., Haberland, V., Baird, D., Walker, V., Haycock, P. C., Hurle, M. R., Gutteridge, A., Erola, P., Liu, Y., Luo, S., et al. (2020). Phenome-wide mendelian randomization mapping the influence of the plasma proteome on complex diseases. *Nature genetics*, *52*(10), 1122–1131.
