# Supplementary material for: Mendelian randomization analysis to identify potential drug targets for osteoarthritis
Source: PLoS One. 2025 Feb 11;20(2):e0316824. doi: 10.1371/journal.pone.0316824 (PMC11813149; doi:10.1371/journal.pone.0316824)
Supplement: Attachment — Submitted filename: review.docx [file pone.0316824.s008.docx]

Comments to the authors:
The study is interesting and adds relevant information on the subject, however I would suggest some minor changes to improve readability and understanding. Below are specific suggestions and comments:

1. I would suggest revising the title to use 'Mendelian randomization' for consistency, ensuring that the terminology throughout the manuscript is uniform.
2. Please add a complete STROBE-MR checklist, demonstrating that the study is clearly reported and that it has a strong scientific basis.
3. Line 47. The statement *“However, the pathogenesis of OA has not been fully elucidated and no drugs have been successfully developed to slow its progression”* is valid but could be strengthened by citing recent studies or reviews that support this claim. Highlighting the current gaps in research could further emphasize the importance of exploring new mechanisms and drug targets.
4. Line 92. **"et al"** should include a period.
5. Line 122. There appears to be a formatting error in the sentence: **"Variables from the preliminary analysis. For this..."**. Specifically, there is a missing space before **"For this"**.
6. Line 124,125. In the sentence **"In cases where these five methods yielded inconsistent results, we considered the results from the MR-IVW method as the reference,"** it is unclear why the MR-IVW method was chosen as the reference when inconsistencies occurred. Please provide additional explanation or justification for why the MR-IVW method was selected as the primary approach in these situations.
7. In Mendelian randomization analysis, it is important to test for heterogeneity and horizontal pleiotropy, especially the latter, as the presence of horizontal pleiotropy can affect the reliability of the results. Did the authors conduct these tests? If so, please include the results. If not, please provide an explanation for why these tests were not performed.
8. Line 141. There appears to be a formatting error in the sentence: **"** **revealing the associations between identified plasma pQTLs and other traits"**. Specifically, there is a missing space before **"For SNPs"**.
9. For the Materials and Methods section regarding phenotype scanning, I suggest attaching the search results as a supplementary file. I believe this will better support your findings.
10. Regarding lines 165-166, is this **P < 5 × 10⁻⁵** correct, and does it conflict with the methods section mentioned earlier? Please review your analysis. If **P < 5 × 10⁻⁵** is applied, what is the reference or basis for this threshold? Please provide a detailed explanation.
11. Line 173. There should be a space between 'Knee' and 'OA' in the phrase 'Within KneeOA'.
12. Line 175. There should be a space between 'Hip' and 'OA' in the phrase 'Within HipOA'.
13. Line 177. There should be a space between 'Finger' and 'OA' in the phrase 'Within FingerOA'. Please check if there are any other similar cases and correct them as well.
14. Line 259. 'SspineOA' should be corrected to 'spine OA'.
15. For section 3.2 'Causal proteins in five types of OA,' which analysis method was applied as the main method? Please display this in Table 2. In section 2.4 'Mendelian randomization analysis,' you described that a single SNP applies the Wald ratio, while multiple SNPs apply the IVW method. However, it appears that only the Wald ratio method was used. Please provide a detailed description of the exact MR method applied at each step in the Methods section and explain the rationale.
