# Supplementary material for: Mendelian randomization analysis to identify potential drug targets for osteoarthritis
Source: PLoS One. 2025 Feb 11;20(2):e0316824. doi: 10.1371/journal.pone.0316824 (PMC11813149; doi:10.1371/journal.pone.0316824)
Supplement: Attachment — Submitted filename: Response to Reviewers.doc [file pone.0316824.s012.doc]

**Dear Editor and Reviewers,**

We would like to express our sincere gratitude to you for taking the time to review and evaluate our manuscript, titled “Mendelian randomization analysis to identify potential drug targets for osteoarthritis” (Manuscript ID: PONE-D-24-36317). We greatly appreciate the detailed and insightful suggestions and comments you have provided to improve the quality of the manuscript. These valuable inputs have helped us further refine the logic and scientific robustness of the study, while also offering opportunities to improve the structure and clarity of the paper.

We fully recognize that the meticulous work of the editor and reviewers is essential in ensuring the rigor and academic quality of the research. Your constructive criticism and professional guidance have been incredibly important to our team, helping us identify areas that may have been overlooked and improve upon them. We sincerely thank you for your suggestions regarding the study design, data analysis, and interpretation of the results. We have carefully considered all the feedback and have provided a point-by-point response to each issue and suggestion raised.

We hope that the revised manuscript will meet with your approval. If there are any further modifications we can make, we would be more than happy to address them in order to improve the manuscript.

Thank you again for your support and for helping us improve our work. We look forward to your continued review and hope the revised version meets the journal’s requirements.

Please don’t hesitate to contact me if you have any questions.

Yours sincerely,

**Corresponding author:**

Xiaochun Wei (sdeygksys@163.com)

Xueqin Rong (13912048078@163.com)

**Response to the Editor**

**1、Sharing Code**

Thank you for your guidance regarding code-sharing requirements. We have uploaded the code underlying the results in this manuscript to the Figshare database to support reproducibility for other researchers. The code is now publicly available with a DOI, along with accompanying documentation and licensing information. All relevant materials comply with the guidelines of PLOS ONE. Link: https://doi.org/10.6084/m9.figshare.27612936.v2.

**2、The role of funders in research**

Dr. Xiaochun Wei, the principal investigator of the National Natural Science Foundation of China (grants U23A6009, U21A20353, and 82172503) and the Shanxi Key R&D Program (grant 202202040201012), provided valuable insights and revisions to the research content. Dr. Xueqin Rong, the principal investigator of the Hainan Provincial Medical and Health Research Program (grant 21A200349), reviewed and edited the manuscript. Dr. Pengcui Li, the principal investigator of the Natural Science Foundation of Shanxi Province (grant 20210302123285), contributed to revising the framework and content of the manuscript.

**3、Response to Data Availability Links for Manuscript**

Thank you for your guidance on providing more specific data availability links. We have updated the information as follows:

- The plasma pQTL data used for preliminary Mendelian randomization analysis can be accessed directly at the following link: https://www.decode.com/summarydata/.
- The plasma pQTL data used for external validation were obtained from Supplementary Table S1 in the original author’s article (https://pmc.ncbi.nlm.nih.gov/articles/instance/7610464/bin/EMS118445-supplement-Supplementary_Tables.xlsx), after applying a selection process on Table S1.
- The osteoarthritis GWAS data can be accessed directly at https://msk.hugeamp.org/downloads.html, where the relevant downloads can be found.

We hope this resolves the issue, and please feel free to reach out if further modifications are needed.

**Response to the Reviewers**

**Reviewer 1**

The study is interesting and adds relevant information on the subject, however I would suggest some minor changes to improve readability and understanding. Below are specific suggestions and comments:

1. I would suggest revising the title to use 'Mendelian randomization' for consistency, ensuring that the terminology throughout the manuscript is uniform.

Response: Thank you for your suggestion. We have carefully revised the title to ensure consistency in the use of the term "Mendelian randomization" throughout the manuscript. The updated title is now: “Mendelian randomization analysis to identify potential drug targets for OA.” We have also reviewed the entire manuscript to maintain uniformity in terminology.

2. Please add a complete STROBE-MR checklist, demonstrating that the study is clearly reported and that it has a strong scientific basis.

Response: Thank you for pointing this out. We had uploaded the STROBE-MR checklist during the initial submission, but to ensure it is easily accessible, we have reorganized the checklist and will re-upload it with the revised submission. This will help demonstrate the clarity and scientific robustness of our study. We appreciate your attention to this important detail.

3. Line 47. The statement *“However, the pathogenesis of OA has not been fully elucidated and no drugs have been successfully developed to slow its progression”* is valid but could be strengthened by citing recent studies or reviews that support this claim. Highlighting the current gaps in research could further emphasize the importance of exploring new mechanisms and drug targets.

Response: Thank you for this valuable suggestion. To strengthen the statement and better support our claim, we have incorporated two recent studies (PMID: 39145815 and 39371059) that discuss the ongoing challenges in understanding OA pathogenesis and the lack of effective drugs to slow its progression. These references highlight the current gaps in OA research, further emphasizing the need for exploring new mechanisms and potential drug targets.

Additionally, we have updated the reference list and reordered the citations accordingly in the manuscript. We believe these revisions provide a stronger foundation for our argument and improve the overall scientific rigor of the paper.

4. Line 92. **"et al"** should include a period.

Response: Thank you for your keen observation. We acknowledge that "et al." was missing a period in Line 92. We have corrected this oversight and ensured that "et al." is appropriately formatted throughout the manuscript. Thank you for bringing this to our attention.

5. Line 122. There appears to be a formatting error in the sentence: **"Variables from the preliminary analysis. For this..."**. Specifically, there is a missing space before **"For this"**.

Response: Thank you for pointing out this formatting error. We have corrected the sentence in Line 130 by adding the missing space before "For this." The revised sentence now reads clearly and maintains proper formatting. We appreciate your meticulous review, which helps us enhance the quality of the manuscript.

6. Line 124,125. In the sentence **"In cases where these five methods yielded inconsistent results, we considered the results from the MR-IVW method as the reference,"** it is unclear why the MR-IVW method was chosen as the reference when inconsistencies occurred. Please provide additional explanation or justification for why the MR-IVW method was selected as the primary approach in these situations.

Response: Thank you for raising this important point. The MR-IVW method was chosen as the reference in cases of inconsistent results because it is considered the most statistically powerful approach when all instrumental variables (SNPs) meet the core assumptions of Mendelian randomization. Specifically, the MR-IVW method assumes no pleiotropy and is widely regarded as providing the most reliable estimates when the majority of SNPs are valid instruments.

We have now added a more detailed explanation in the manuscript to clarify this choice, emphasizing that the MR-IVW method was selected due to its higher power and more precise estimates under the assumption of no horizontal pleiotropy. The revised text in Lines 133-140 now provides clearer justification for using MR-IVW as the primary approach in these cases.

7. In Mendelian randomization analysis, it is important to test for heterogeneity and horizontal pleiotropy, especially the latter, as the presence of horizontal pleiotropy can affect the reliability of the results. Did the authors conduct these tests? If so, please include the results. If not, please provide an explanation for why these tests were not performed.

Response: Thank you for raising this important point regarding heterogeneity and horizontal pleiotropy testing in MR. In our analysis, we did not perform formal heterogeneity and pleiotropy tests because we had only one significant SNP. In the case of a single SNP, methods like MR-Egger or heterogeneity testing are not applicable, as these require multiple SNPs to test for pleiotropic effects or heterogeneity across instruments.

However, to address concerns related to potential horizontal pleiotropy, we conducted a Bayesian colocalization analysis and phenotype scanning. The colocalization analysis helps to ensure that the association between the SNP and OA is driven by the same causal variant, thus reducing the likelihood of pleiotropy. Additionally, the phenotype scan allowed us to check whether the significant SNP is associated with other traits, potentially indicating pleiotropic effects or confounding. We believe this approach provides an alternative means of addressing horizontal pleiotropy in our analysis.

These analyses and results are already included in our manuscript to address the issue of horizontal pleiotropy. We believe this approach effectively strengthens the reliability of our findings.

8. Line 141. There appears to be a formatting error in the sentence: **"** **revealing the associations between identified plasma pQTLs and other traits"**. Specifically, there is a missing space before **"For SNPs"**.

Response: Thank you for your attention to detail. We have corrected the formatting error in Line 160 by adding the missing space before "For SNPs." The revised sentence now reads clearly and maintains proper formatting. We appreciate your feedback, which has helped us enhance the manuscript's clarity.

9. For the Materials and Methods section regarding phenotype scanning, I suggest attaching the search results as a supplementary file. I believe this will better support your findings.

Response: Thank you for your suggestion. We appreciate your recommendation to attach the search results as a supplementary file. We would like to clarify that we had initially uploaded the results as Supplementary Material Table S3, along with other materials, in a compressed file format during our initial submission. To ensure accessibility, we will recheck and confirm that these supplementary materials are clearly labeled and included with the revised manuscript. Your input is valuable in enhancing the clarity and robustness of our findings.

10. Regarding lines 165-166, is this **P < 5 × 10⁻⁵** correct, and does it conflict with the methods section mentioned earlier? Please review your analysis. If **P < 5 × 10⁻⁵** is applied, what is the reference or basis for this threshold? Please provide a detailed explanation.

Response: Thank you for your insightful comment. Upon reviewing the manuscript, we recognize that there was an error in our reporting of the significance threshold. The correct threshold should indeed be P < 5 × 10^−8^, consistent with the criteria mentioned in the Methods section. We apologize for this oversight and have made the necessary corrections in Lines 165-166 to reflect the correct threshold. The revised statement now accurately states the appropriate threshold for significance, ensuring consistency throughout the manuscript. We appreciate your diligence in identifying this discrepancy, which has allowed us to clarify this important aspect of our analysis.

11. Line 173. There should be a space between 'Knee' and 'OA' in the phrase 'Within KneeOA'.
12. Line 175. There should be a space between 'Hip' and 'OA' in the phrase 'Within HipOA'.
13. Line 177. There should be a space between 'Finger' and 'OA' in the phrase 'Within FingerOA'. Please check if there are any other similar cases and correct them as well.
14. Line 259. 'SspineOA' should be corrected to 'spine OA'.

Response: Thank you for your careful review and for identifying these formatting issues. We have made the necessary corrections as follows:

- Added a space between "Knee" and "OA" to read "Within Knee OA."
- Added a space between "Hip" and "OA" to read "Within Hip OA."
- Added a space between "Finger" and "OA" to read "Within Finger OA."
- Corrected "SspineOA" to "spine OA".

Additionally, we have conducted a thorough review of the manuscript to ensure there are no other similar formatting issues present. We appreciate your attention to detail, which has helped us improve the clarity and presentation of the manuscript.

15. For section 3.2 'Causal proteins in five types of OA,' which analysis method was applied as the main method? Please display this in Table 2. In section 2.4 'Mendelian randomization analysis,' you described that a single SNP applies the Wald ratio, while multiple SNPs apply the IVW method. However, it appears that only the Wald ratio method was used. Please provide a detailed description of the exact MR method applied at each step in the Methods section and explain the rationale.

Response: Thank you for your helpful suggestion regarding the clarity of the analysis methods in Section 3.2 and the explanation of MR methods in Section 2.4.

To address your comment, we have made the following updates:

- Table 2 Update: We have added a new column labeled "Method" in Table 2 to clearly indicate the specific MR method used for each analysis. This will help readers easily identify whether the Wald ratio or IVW method was applied for each protein and OA type.
- Explanation of Wald Ratio and IVW Methods: In our study, when only one SNP was available as the instrument, we applied the Wald ratio method. This method is suitable for single SNP analyses, as it directly estimates the causal effect by dividing the SNP-outcome association by the SNP-exposure association. For analyses involving multiple SNPs, we applied the IVW method. The IVW method combines the effect estimates from each SNP using a meta-analysis approach, assuming that all SNPs are valid instruments. It provides a more robust estimate when multiple independent SNPs are used as instruments, allowing for greater statistical power and accounting for variability in individual SNP estimates.

We have revised the Methods section to reflect these updates, including detailed descriptions of why each method was used. This will clarify the rationale behind our choice of methods in different analysis scenarios.(Lines 116-123)

We appreciate your feedback and believe these modifications will enhance the clarity and transparency of our methodology.

**Reviewer 2**

**Comments**

• In Section 2.2, p.3, line.95, the citations for the OA GWAS data (Citation 12 and 13) may not be accurate. The correct reference appears to be Boer et al. (2021). Additionally, the description stating " a large-scale meta-analysis of GWAS in the UK Biobank" (p.3, Ins. 93-94) can be misleading, as the UK Biobank is only one of the cohorts included in the meta-analysis (Boer et al., 2021).

Response: Thank you for your comment. We have corrected the citations for the OA GWAS data in Section 2.2. The original references (Citations 12 and 13) were not primary sources, and we have now replaced them with the correct reference to Boer et al. (2021). Additionally, We also recognize that our description of the GWAS meta-analysis was incomplete. The UK Biobank is only one of the nine cohorts included in the analysis, and we have revised the text accordingly in the manuscript.（lines 94-97）

• Is there sample overlap between the protein and OA GWAS? For example, one of the 9 cohorts in the OA meta-analysis is Icelandic (cohort name: deCODE) (Boer et al., 2021), while the protein GWAS also utilizes Icelandic samples. It would be important to verify whether these represent two different Icelandic cohorts. Additionally, since the OA GWAS includes individuals from the UK Biobank, do any of the five protein GWAS studies (listed in Citation 11) used for external validation also involve UK Biobank participants? Given that two-sample MR generally requires non-overlapping samples for the exposure and outcome, it is essential to investigate any potential sample overlap.

Response: Thank you for your thoughtful question. We fully acknowledge the concerns you raised regarding potential sample overlap. Indeed, two-sample MR assumes that the exposure and outcome come from independent samples, and any sample overlap can impact the accuracy of causal inference results.

First, regarding the Icelandic cohort you mentioned, while it is true that the OA GWAS analysis includes the Icelandic deCODE cohort, based on our current data, the Icelandic samples in the protein GWAS and OA GWAS do not come from the same population. We will further verify this to ensure that the samples used in these studies are independent. In addition, your point about the UK Biobank samples is important. While some UK Biobank samples were indeed used in the protein GWAS, there is not a complete overlap with the OA GWAS samples. To ensure the robustness of our analysis, we plan to use LD score regression (LDSC) to assess any potential sample overlap.

LDSC provides important insights through the genetic correlation coefficient (rg) and SNP heritability (h²) estimates. We will focus on the following key points:

1. Genetic correlation coefficient (rg) and its p-value: If the rg is small (typically |rg| ≤ 0.2) and the p-value is non-significant (p > 0.05), we can reasonably assume that there is no significant genetic overlap between the samples. A small rg value suggests that the traits are genetically uncorrelated, helping to rule out the possibility of sample overlap.

2. SNP heritability (h²): If the h² estimates for each trait fall within a reasonable range (0.05–0.2) and show no significant discrepancies between them, this further supports the independence of the samples.

Our analysis shows that the genetic correlation coefficients between the four identified proteins and OA are |rg| ≤ 0.2, with non-significant p-values (p > 0.05). Therefore, we can reasonably conclude that the samples used in our study are independent, and the impact of sample overlap is minimal.

We will include these results in our final manuscript and appreciate your attention and thoughtful feedback on our research.

| **Proteins** | **h**^2^ | **h2_SE** | **RG** | **RG_SE** | **RG_P** |
| --- | --- | --- | --- | --- | --- |
| DNAJB12 | 0.0766 | 0.0192 | 0.0876 | 0.0735 | 0.2337 |
| USP8 | 0.0395 | 0.0147 | 0.0985 | 0.0834 | 0.2379 |
| IL12B | 0.0985 | 0.0216 | 0.0149 | 0.0800 | 0.8523 |
| RGMB | 0.1204 | 0.0260 | -0.2095 | 0.0937 | 0.1254 |

• In Section 2.3, p.5, line.107, I wonder whether it is necessary to screen SNPs using the F < 10 criterion. For a SNP with an effect size β and standard error SE, the t-statistic is given by t = . In a two-sided t-test, if a SNP passes the threshold of p < 5 ×10^−8^, then it implies that t > 5.45. Since the F statistic is calculated as F = t^2^ , it follows that F > 5.45^2^ > 10. In other words, if the SNP screening procedure using the p < 5 × 10^−8^ criterion is already applied, then all SNPs that pass this criterion would inherently have F > 10. Therefore, it might be worth double-checking why the F statistic is still needed for IV selection after applying the genome-wide significance criterion.

Response: Thank you for raising this point. While it is correct that SNPs passing the genome-wide significance threshold (p < 5×10^−8^) would inherently have an F-statistic greater than 10, we have included the F-statistic criterion for consistency with previous studies and as a safeguard to ensure instrument strength in all contexts. This additional check helps us to confirm robust instrument selection across different datasets and analysis scenarios. Therefore, we believe it is still valuable to explicitly apply this criterion.

• In Section 4, p.9, lines 241-243, it states that "we only utilize cis-pQTLs as instruments." However, there is no related description in the IV selection section (Section 2.3), nor is there any information on how the cis-pQTLs are selected. For example, in Citation 11, the authors define cis-acting pQTLs within a 500-kb window on either side of the leading pQTL of the protein (Zheng et al., 2020). How is this selection process conducted in this study?

Response: Thank you for pointing this out. We acknowledge that the selection process for cis-pQTLs was not fully described in Section 2.3. In our study, we focused on cis-pQTLs because they are more likely to directly influence the expression of the corresponding proteins, thereby minimizing the risk of pleiotropy and confounding effects compared to trans-pQTLs. Consistent with Zheng et al. (2020), we defined cis-pQTLs as variants located within a 500-kb window on either side of the leading pQTL for each protein. We will revise Section 2.3 to include a detailed explanation of this selection process and the rationale for focusing on cis-pQTLs in the updated manuscript.（lines 102-105）

• In Citation 11, the authors filtered out SNPs associated with more than five proteins as they might be highly pleiotropic, see the Methods section in Zheng et al. (2020). I wonder if this study conducted a similar procedure. If not, it might be helpful to provide information on the number of associated proteins for each pQTL. For example, the histogram in Panel A of Supplementary Figure 6 in Zheng et al. (2020) illustrates this well.

Response: Thank you for your question. After filtering for significant pQTLs in our analysis, we found that these significant pQTLs were associated with a maximum of only three proteins. Therefore, we did not perform a procedure similar to that in Citation 11, which involves removing pQTLs associated with more than five proteins, as none of the significant pQTLs in our data were associated with more than five proteins. We believe this enhances the reliability of our analysis results.

• In Section 2.6, p.5, line.134, should "share a single variant" be revised to "share a single causal variant"? Additionally, in line 135, it would be helpful to clarify what hypothesis 4 is.

Response:Thank you for your suggestion. We have revised "share a single variant" to "share a single causal variant" to more clearly indicate that we are discussing potential causal relationships rather than merely shared variants. In the text, we indeed lack a specific description of hypothesis 4. Hypothesis 4 refers to the "causal relationship hypothesis," which states that the association between phenotype 1 and phenotype 2 is driven by a shared causal variant within a specific biological and genetic context. We will provide a detailed explanation of this hypothesis to enhance the clarity of the text.(lines 150-153)

• In Section 2.6, p.5, lines 135-136, it states, "In PPH4, we examine...the shared variant region." How was the variant region defined? There is no related description provided.

Response:Thank you for your valuable comment. In the colocalization analysis, we defined the variant region as ±500 kb around the significant plasma pQTL locus. This region was chosen to ensure that potential causal variants are included, enhancing the accuracy of the colocalization analysis. We have added this clarification to Section 2.6 in the revised manuscript to explain how the variant region was defined.(lines 153-156)

• In Section 2.7, p.5, lines 145-146, what is meant by "priority protein pQTLs"? Addi- tionally, are the results of the LD analysis presented anywhere in the paper? If so, please provide a reference; if not, please include them.

Response:Thank you for your question. Regarding the term "priority protein pQTLs," we refer to the significant pQTLs identified in our study, which are believed to play an important role in the association between proteins and disease phenotypes. Additionally, We acknowledge that the statement regarding calculating the LD between the significant pQTLs we identified for priority proteins was misleading. This phrase was intended to indicate that the linkage disequilibrium among the pQTLs we filtered was low. However, since each protein is associated with only one significant pQTL, there is no need for LD analysis. Therefore, we have removed this statement in the revised manuscript.

• In Section 3.2, p.6, line.172, it states that the Bonferroni adjusted p-value threshold is 0.05/1553, as there are 1553 proteins involved in the analysis for each OA trait. However, Supplementary Table 2 presents fewer than 1553 proteins with estimates. What is the reason for this discrepancy? Is it because fewer than 1553 proteins are used for assessing causality for each OA trait? If so, should the Bonferroni p-value threshold be adjusted accordingly? For example, for knee OA, there are 1164 proteins used for causal estimation, should the p-value threshold adjusted to 0.05/1164?

Response:Thank you for your comments and suggestions. I would like to clarify the reason for the number of proteins in Supplementary Table 2 being less than 1553:

The number of proteins in Supplementary Table 2 is less than 1553 because not all pQTLs have corresponding SNPs for each OA trait, which results in fewer available proteins for actual causal estimation than 1553. Regarding the Bonferroni adjusted p-value threshold, we determined a total of 1553 proteins as the targets for preliminary analysis during the study design phase. Therefore, the Bonferroni adjusted p-value threshold is calculated based on this total number, specifically 0.05/1553. The fundamental principle of Bonferroni correction is to reduce the overall significance level (e.g., 0.05) by dividing it by the number of comparisons (in this case, 1553) to effectively control the first-type error rate. This helps ensure that the probability of incorrectly rejecting the null hypothesis during multiple hypothesis testing is minimized. Although fewer proteins (e.g., 1164) are used in some analyses, the Bonferroni adjusted threshold remains applicable. This is because the adjustment is designed to account for all potential hypothesis tests in the preliminary analysis. Maintaining this initial p-value threshold helps ensure the conservativeness and consistency of statistical results, thereby reducing the false discovery rate associated with multiple comparisons. In summary, we will keep the Bonferroni adjusted p-value threshold at 0.05/1553 to ensure the scientific rigor and statistical reliability of the results.

• In Section 3.3, p.7, line.186, what is the reason for the absence of reverse MR results for IL12B?

Response:Thank you for your question. I would like to clarify the reason for the absence of reverse MR results for IL12B: In the reverse MR analysis, there is only one significant SNP for Spine OA. However, this significant SNP does not have a corresponding SNP in the IL12B data, which prevents us from using the harmonise_data() function to merge the data. As a result, due to the lack of matching SNPs, we are unable to conduct causal analysis.

• In Section 3.3, p.7, line.188, "causal relationship between OA and the respective screened proteins" should be revised to "causal relationship from OA to the respective screened proteins" to indicate it is the reverse causal diretcion.

Response:Thank you for your suggestion. We will revise the sentence from "causal relationship between OA and the respective screened proteins" to "causal relationship from OA to the respective screened proteins" to clearly indicate the direction of the reverse causal relationship.(lines 215)

• It would be helpful to include the full estimation results of the reverse MR using all five MR methods, as well as the complete results of the external validation, in the supplementary materials.

Response: Thank you for your suggestion. We will include the full results of the reverse MR analysis and external validation in the supplementary materials. The reverse MR results will be uploaded as Table S3, and the complete external validation results will be uploaded as Table S5. These supplementary materials will provide readers with more detailed estimates and validation data to further understand our findings.

• Some references seem to be absent. For example, additional references could be in- cluded in the Introduction when defining OA (p. 2, lines 43-44), discussing the trend in OA prevalence (p. 2, lines 46-47), mentioning the statement "Dysregulation of the human plasma proteome... as is the case with OA" (p. 2, lines 53-54), and defining MR (p. 2, lines 61-63). Additionally, what are the references for MR-IVW and the four MR methods used in the reverse causality analyses (MR-Egger, weighted median method, simple mode, and weighted mode)? Also, what are the references for Steiger filtering and the Bayesian co-localization method?

Response: Thank you for pointing out the issue with missing references. We have addressed your comments by adding the relevant references in the revised manuscript, including those related to the definition of OA, trends in OA prevalence, the relationship between dysregulation of the plasma proteome and OA, the definition of Mendelian randomization (MR), the various MR methods used in reverse causality analyses (such as MR-IVW, MR-Egger, weighted median, etc.), as well as the relevant literature on Steiger filtering and Bayesian co-localization analysis.

**• Clarification questions:**

1. In Abstract, p.1, line.20, should "Causal associations between 4907 circulating proteins and five OA-related traits...." be revised to "....between 1553 circulating proteins....", as after the IV selection procedure, only 1553 out of 4907 proteins are selected for MR analyses.

Response: Thank you for pointing out this issue. In the revision, we have corrected "4907 circulating proteins" to "1553 circulating proteins" to reflect that, after applying the significant IV selection criteria, only 1553 proteins have significant pQTLs. This change ensures that the description in the abstract is consistent with our analysis results.(lines 20)

2. In Abstract, p.1, lines 23-24, the statement "....inverse MR analysis and Steiger filtering to ensure positive associations between circulating proteins and OA" does not seem accurate. Why is it stated "to ensure positive associations"? My understanding is that these analyses are primarily used to assess the direction of causality. A similar statement appears in Section 2.5, p.5, In.127. "....Steiger filtering as a dual test to ensure a positive association....".

Response: Thank you for the valuable feedback. Our wording was indeed inaccurate. It is correct that inverse MR analysis and Steiger filtering are used to assess causal relationships. What we intended to convey is that our goal is to ensure that the direction of the causal relationship is from proteins to OA, while there should not be a causal relationship in the reverse direction, i.e., from OA to proteins. Therefore, we conducted these two analyses. We will revise the text to reflect this clarification more accurately.(lines 23-24)

3. In Abstract, p.1, line.25, what does "cascading effects" refer to?

Response: Thank you for your question. The use of "cascading effects" here may not have been entirely accurate. Our intention was to refer to the elimination of potential confounding effects or horizontal pleiotropy through Bayesian co-localization and phenotypic scanning, rather than a series of causal chain effects. Therefore, we will revise the manuscript to replace "cascading effects" with "confounding effects" to ensure clarity and precision.(lines 25)

4. In Section 2.3, p.4, line.103, what is meant by "chain imbalance"?

Response: Thank you for pointing this out. The term "chain imbalance" was a translation error. The correct term should be "linkage disequilibrium." We will correct this mistake in the revised version to ensure accurate terminology.(lines 106)

5. In Section 2.3, p.4, line.105, is "harmonise_data()" a function from the R package "TwoSampleMR"? It would be helpful to provide full details.

Response: Thank you for your question. The "harmonise_data()" function is indeed from the R package "TwoSampleMR." We have already provided more detailed information in the revised manuscript to ensure completeness.(lines 108-109)

6. In Section 2.4, p.4, line.115, what does "heterogeneity analysis" refer to? Does it refer to the analysis using the four MR methods in addition to MR-IVW?

Response: Thank you for your question. Heterogeneity analysis is typically an important step in causal inference studies and is used to assess consistency across different instrumental variables. However, in our study, each protein has only one significant pQTL, which makes it impossible to conduct a heterogeneity analysis. Such analysis usually requires multiple instrumental variables (SNPs), but in this case, the condition is not met.

7. In Section 2.5, p. 5, line.128, what is meant by "SNP mismatch"? Does it refer to SNPs that influence the outcome (and are therefore correlated with the exposure) being used as IVs for the exposure?

Response: Thank you for your question. Both inverse MR analysis and Steiger filtering are primarily used to rule out reverse causality. In our inverse MR analysis, there was only one significant SNP for Spine OA, and this SNP did not correspond to one in IL12B, which made it impossible to conduct the inverse MR analysis. Therefore, we used Steiger filtering as a supplementary analysis. The term "SNP mismatch" refers to the fact that the Spine OA SNP could not be matched in IL12B. The manuscript has been revised accordingly.(lines 142-143)

8. In Section 2.8, p. 5, line.149, what is meant by "as a new exogenous exposure"? Does this refer to using the new proteins from Citation 11 as exposures? If so, the term "exogenous" could be misleading, as all the exposures in this context are endogenous and therefore require IVs for causal inference.

Response:Thank you for your question. Here, "as a new exogenous exposure" refers to our use of plasma protein data from other researchers to conduct a re-analysis with our OA to validate the initial MR results obtained using the protein data. We acknowledge that the use of the term "exogenous" could be misleading, as all exposures in this context are endogenous and indeed require IVs for causal inference. We will correct this wording in the revised manuscript to avoid confusion. (lines 167)

9. In Section 3.3, p. 7, line 184, the title of the subsection mentions "five causal proteins." Why is it five?

Response: Thank you for pointing this out, and we apologize for the oversight. The subsection title was intended to refer to the sensitivity analyses conducted on the causal relationships identified across the five OA traits, rather than being limited to five causal proteins. We will correct this title in the revised manuscript to avoid confusion.(lines 211)

10. In Section 3.3, p. 7, lines 202-203, what is meant by "associations with themselves"?

Response: Thank you for your question. "Associations with themselves" refers to the significant pQTLs of the proteins DNAJB12, USP8, RGMB, and IL12B being only related to their respective proteins. For example, the significant pQTL for DNAJB12 is rs9416018, meaning that rs9416018 should only be strongly associated with DNAJB12 and not with other factors influencing OA, such as bone density. This analysis is conducted to satisfy the third assumption of MR (to exclude confounding factors).(lines 227-231)

11. In Section 6, p.12, line.347, why are the four causal proteins described as "strongly correlated"? If this refers to these proteins having significant estimates, the use of "correlated" could be misleading, as the final conclusion is that the proteins are likely to exert causal effects, rather than simply exhibiting correlations.

Response: Thank you for your helpful suggestion. We acknowledge that the use of the term "strongly correlated" was inappropriate. In this context, we were referring to the significant estimates of these proteins in our causal analysis, aiming to highlight their potential causal relationships rather than mere correlations. Therefore, we will revise "strongly correlated" to "significant causal relationships" to eliminate any potential confusion.(lines 379-380)

• The sheet names in Supplementary Table S1 are not in English.

Response: Thank you for your feedback. We will ensure that the sheet names in Supplementary Table S1 are in English to improve readability and international accessibility

We sincerely thank the editors for our appreciation and evaluation of this paper, and we sincerely appreciate the reviewers for guiding us to improve the quality of the manuscript!

Please don’t hesitate to contact me if you have any questions.

Best wishes,

Xiaochun Wei，and Xueqin Rong
