# Supplementary material for: Mendelian randomization analysis to identify potential drug targets for osteoarthritis
Source: PLoS One. 2025 Feb 11;20(2):e0316824. doi: 10.1371/journal.pone.0316824 (PMC11813149; doi:10.1371/journal.pone.0316824)
Supplement: Attachment — Submitted filename: Response to Reviewers (2).doc [file pone.0316824.s014.doc]

**Dear Editor and Reviewers,**

We would like to sincerely thank you for your thoughtful and constructive feedback on our manuscript titled "Mendelian Randomization Analysis to Identify Potential Drug Targets for Osteoarthritis" (Manuscript ID: PONE-D-24-36317) during the second round of review. We greatly appreciate the time and effort you have dedicated to evaluating our manuscript once again and providing valuable suggestions to further enhance its quality.

We have carefully addressed all the comments and suggestions raised in the second review and made the necessary revisions to improve the manuscript. Specifically, we have incorporated the feedback regarding references, clarifications on the methods, and adjustments to phrasing to ensure the manuscript’s clarity, accuracy, and scientific rigor. Additionally, we have ensured that all points raised have been addressed thoroughly in our revised manuscript.

We believe the changes made in response to your feedback have significantly strengthened the manuscript, and we hope it now meets the expectations and requirements of the journal. Should there be any further areas that require clarification or additional revisions, we would be more than happy to address them.

Once again, thank you for your valuable support and guidance in improving our work. We look forward to your feedback on the revised manuscript and hope it meets your approval.

Please do not hesitate to contact me if you have any questions or need further clarification.

Yours sincerely,

**Corresponding author:**

Xiaochun Wei (sdeygksys@163.com)

Xueqin Rong (13912048078@163.com)

**Response to the Reviewers**

**Further Comments**

1. In the corrected citation for the OA GWAS (i.e., Boer et al. (2021)), the authors indicate that their GWAS identified risk variants for 11 OA-related phenotypes (as mentioned in the Summary section and the Graphical Abstract of the paper). However, in the revised manuscript, it is stated that the study involved “826,690 individuals (including 177,517 OA patients) and covered 12 OA-related traits” (p.3, line 96). Could you clarify why 12 OA traits are mentioned here, rather than 11 as stated in the cited paper? Additionally, please revise Figure 1B to reflect the correct reference for the OA data: Under “Outcome: Osteoarthritis,” it currently lists “Tachmazidou et al. (2019)” instead of “Boer et al. (2021)”. Also, in Figure 1B, under “Exposure: Proteins,” it might be misleading to state “35,559 controls,” as proteins are not case-control phenotypes. It may be more accurate to describe this as “35,559 participants.”

Response: Thank you for your insightful comments. We have carefully reviewed and addressed each point as follows:

1)、Clarification of the number of OA-related phenotypes: We appreciate your observation regarding the discrepancy in the number of OA-related phenotypes. In Boer et al. (2021), the authors explicitly mention 11 OA-related phenotypes. The mention of 12 OA-related traits in our manuscript was an oversight on our part, and we have now corrected this to reflect the accurate number, i.e., 11 OA-related phenotypes, as reported in the cited paper.（line 95）

2)、Correction of reference in Figure 1B (Outcome: Osteoarthritis): We acknowledge the error in the reference cited in Figure 1B. The correct reference for the OA data should indeed be “Boer et al. (2021)” instead of “Tachmazidou et al. (2019).” We have updated Figure 1B to reflect this correction.

3)、Revision of “35,559 controls” to “35,559 participants” in Figure 1B (Exposure: Proteins): Thank you for pointing out the potential for misunderstanding. We agree that describing the dataset as “35,559 participants” more accurately reflects the nature of the protein data. We have revised Figure 1B accordingly.

All changes have been incorporated into the revised manuscript and accompanying figures. We sincerely appreciate your detailed review and constructive feedback, which have improved the clarity and accuracy of our work.

2. Further comment: In the protein GWAS paper by Ferkingstad et al. (2021), the Methods section states: “We collected plasma samples from 40,004 Icelanders through two main projects: the Icelandic Cancer Project (52% of participants; samples collected from 2001 to 2005) and various genetic programs at deCODE genetics, Reykjavík, Iceland (48%).” In the OA GWAS paper by Boer et al. (2021), the authors also indicate that one of the cohorts used for meta-analysis is deCODE (refer to the Key Resources table in the paper). Given this overlap, could you please provide solid evidence supporting your claim that “the Icelandic samples in the protein GWAS and OA GWAS do not come from the same population”?.......

Response: Thank you for your detailed review and valuable suggestions regarding the use of LDSC analysis in our study. We greatly appreciate your feedback, and have made the following revisions accordingly:

- Regarding sample overlap: We have applied the cross-trait LDSC method proposed by Bulik-Sullivan et al. to assess sample overlap between proteins and OA, and have reported the intercept values. The results show that the intercept is close to zero, and the difference from zero is not statistically significant, indicating no significant sample overlap between the proteins and OA. In line with your suggestion, we have revised the manuscript to focus on reporting the intercept values rather than genetic correlation or heritability, as the latter is not affected by sample overlap.（line 162-178）
- Regarding the LDSC analysis method: In Section 2.9 of the methods, we have added further details and explicitly stated that the ‘ldsc’ R package was used for the LDSC analysis, with the primary function being ldsc(). This clarification ensures a more transparent description of the analysis steps and procedures.
- Modifications to Sections 2.8 and 3.4: Following your suggestion, we have updated the method in the revised Section 2.9 and the results in Section 3.4, with a focus on reporting the intercept results and highlighting the importance of the intercept in testing for sample overlap.（line 236-248）

Thank you again for your insightful comments. We believe these revisions will enhance the clarity and accuracy of the manuscript. If you have any further questions or suggestions, we would be happy to continue the discussion.

3. Further comment: If there are no specific reasons for including the F > 10 criterion in the IV selection process, I do not believe it is necessary to retain this step. To my knowledge, it is standard practice in MR studies to apply only the genome-wide significance threshold (p < 5 × 10−8 ) to ensure instrument strength, as the F > 10 criterion becomes redundant under this threshold. For example, see the instrument selection steps described in the Methods section of Zheng et al. (2020) (citation 20). I recommend removing the F > 10 step from the IV selection procedure (p.4, lines 110–112) to avoid redundancy and align with standard practices.

Response: Thank you for pointing out the redundancy of including the F>10 criterion in the instrumental variable (IV) selection process. After careful consideration, we agree that this step is unnecessary under the genome-wide significance threshold (p < 5×10^−8^ ), which sufficiently ensures the strength of the selected instruments.

In line with your suggestion and standard practices in MR studies, such as those described in Zheng et al. (2020), we have removed the F>10 criterion from the IV selection process (p.4, lines 110–112). This change ensures consistency with the literature and simplifies the methodology without compromising the validity of the analysis.

The revised manuscript reflects this modification, and we have ensured that the methodology section aligns with this update. Thank you for your valuable feedback in helping us improve the clarity and rigor of our study.

4. I would like to clarify that the Bonferroni adjusted p-value should be based on the total number of (independent) tests performed, rather than the nominal number of exposure phenotypes. If you are treating each set of MR analyses corresponding to each OA trait separately (and ignore the possible correlations between the proteins), the Bonferroni adjusted p-value should be calculated as 0.05 divided by the actual number of proteins used as exposures for that specific OA trait. For example, for knee OA, it should be 0.05/1164 rather than 0.05/1553. Alternatively, if you intend to account for all hypothesis tests jointly by applying a universal Bonferroni corrected p-value, you may need to consider the total number of tests across all OA traits. For example, when testing pairwise causality between 100 exposure traits and 90 outcome traits, the universal Bonferroni p-value would be 0.05/(100×90), resulting in a more conservative threshold. As a reference, it seems that Zheng et al. (2020) (citation 20) applied this type of Bonferroni correction, leading to a stringent threshold of p < 3.5 × 10^−7^ . This threshold is calculated as 0.05/142, 857 where 142, 857 is the total number of (independent) tests between all protein exposures and all outcome traits. This is considerably stricter than 0.05/1002 where 1002 is the number of protein exposures involved in their MR analyses. For more details and complications with defining the number of independent tests, see the subsection “Estimating the causal effects of proteins on human phenotypes using MR” in the Methods section of their paper. I would not insist on revising the current threshold p-value of 0.05/1553, as applying the more lenient OA-specific p-value (e.g., 0.05/1164 for knee OA) does not affect the significance of the current causal relationships. However, this might be an important point to consider in future research.

Response: Thank you for your valuable suggestion regarding the Bonferroni correction. We appreciate your detailed explanation and understand the importance of accurately adjusting for multiple comparisons. However, we would like to clarify the rationale for retaining our current approach:

1)、Independent Analysis of OA Subtypes: Our study analyzed the causal relationships for each OA subtype separately. Since these analyses were conducted independently rather than pooled or combined, applying a universal Bonferroni correction across all OA traits, or recalculating thresholds specific to each OA subtype (e.g., 0.05/1164 for knee OA), may not be strictly necessary in this context. We aimed to maintain consistency in the threshold (0.05/1553) across all analyses to simplify interpretation and align with our study design.

2)、Applicability of Global Correction: The use of a universal Bonferroni threshold is typically employed in studies where all exposure-outcome pairs are analyzed jointly. In our study, each OA subtype was treated as a separate outcome, so a global correction was not considered essential.

3)、No Impact on Results: As noted, the proposed adjustment to subtype-specific thresholds does not affect the significance of our findings. Maintaining the current approach ensures interpretability and consistency while preserving the robustness of our conclusions.

5. Thank you for the clarification. It would be helpful to also include an explanation in the main text, stating that this is due to a lack of data (Section 3.3, p.8, lines 213–214).

Response: Thank you for your helpful suggestion. We have now included an explanation in the main text (Section 3.3, p.8, lines 207–213) to clarify that the absence of reverse MR results for IL12B is due to a lack of data. Specifically, as noted, there is only one significant SNP for Spine OA, but this SNP does not correspond to any SNP in the IL12B dataset, preventing us from using the harmonise_data() function to merge the data. Without matching SNPs, we were unable to perform the reverse MR analysis.（line 211-217）

We hope this clarification improves the readability and understanding of this section.

6. Thank you for providing the results. In Table S3, Column A, the IDs of the results are not in consecutive order, please revise them. In Table S5, Sheet “OR-Knee OA”, Row 3, Column R, there is a number “6.81199E-05”. Please either remove it or clarify its meaning and what it refers to. Additionally, it would be helpful to include the Steiger filtering results in Table S3 as well (in a separate sheet) so that this table provides a comprehensive summary of all the reverse causality results.

Response: Thank you for your helpful feedback. We have made the following revisions in response to your comments:

1）、Table S3, Column A (IDs): We have corrected the IDs in Column A of Table S3 to ensure they are in consecutive order.

2）、Table S5, "OR-Knee OA", Row 3, Column R: The value "6.81199E-05" was inadvertently included during the calculation of the p-value (0.05/734). We have removed it from the table to avoid any confusion.

3）、Steiger Filtering Results: As suggested, we have included the Steiger filtering results in a separate sheet within Table S3, providing a more comprehensive summary of all the reverse causality results.

We appreciate your attention to detail and hope these revisions address your concerns.

7. Thank you for your explanation, which is clear to me. However, I find the phrasing in the manuscript of “to ensure the positive causal relationship between circulating proteins and OA” (line 24 and line 142) to be misleading. It seems to suggest that the sign of the causal effects of the proteins on OA are positive. To avoid confusion, it would be clearer to revise the phrasing to “to ensure the causal direction between circulating proteins and OA.”

Response: Thank you for pointing this out. We agree that the phrasing in the manuscript may cause confusion. To avoid any ambiguity, we have revised the text from “to ensure the positive causal relationship between circulating proteins and OA” to “to ensure the causal direction between circulating proteins and OA” as suggested.（line 24 and line 135）

We appreciate your careful review and hope this revision resolves the concern.

8. A few questions about the referencing.

- In Section 2.4, p.4, lines 122-123, the reference (citation 25) for the statement “This increases statistical power....from multiple independent variants” does not appear to be correct. Could you please clarify which specific part of citation 25 supports this statement? If it does not, please provide a more appropriate reference.
- In Section 2.5, please add references for the statements related to MR-IVW, e.g., “Under the assumption that....precise estimates.” (lines 135-136); “It is also the most statistically powerful....horizontal pleiotropy is absent.” (lines 136-138).
- In Section 2.5, p.5, lines 142-143, the reference for Steiger filtering (citation 25) does not appear to be correct. A more appropriate reference would be Hemani et al. (2017).
- In Section 2.6, p.5, lines 147-150, the reference (citation 29) for Bayesian colocalization does not appear to be correct. Based on the R package used for the colocalization analysis, the appropriate reference for the method seems to be either Giambartolomei et al. (2014) or Wallace (2021). Please further verify this and update the manuscript with the correct reference.
- In Section 2.6, lines 156-157, could you please clarify which specific part of citation 30 supports the posterior probability threshold of 0.8?
- In Section 2.8, p.6, lines 171–172, the reference (citation 32) for LD score regression is not the original source of the method. The correct reference should be Bulik-Sullivan et al. (2015). Please update the citation accordingly.
- I would suggest carefully reviewing the references and citations throughout the manuscript to ensure their accuracy.

Response: Thank you for your detailed suggestions regarding the references in the manuscript. I have thoroughly reviewed and revised the references throughout the manuscript to ensure their accuracy and relevance. Below is a point-by-point response:

1)、Section 2.4, p.4, lines 122-123: Regarding the statement “This increases statistical power… from multiple independent variants,” the original citation has been replaced with a more appropriate reference (now citation 28). This reference more accurately supports the description in this section.

2)、Section 2.5 (MR-IVW method): I have added references related to the MR-IVW method’s assumptions, statistical power, and the absence of horizontal pleiotropy, including citations 28 and 32. Additionally, some vague wording in the original manuscript has been revised for clarity.

3)、Section 2.5, p.5, lines 142-143 (Steiger filtering): The original citation (citation 25) has been replaced with a more suitable reference, Hemani et al. (2017) (now citation 33), as suggested.

4)、Section 2.6, p.5, lines 147-150 (Bayesian colocalization analysis): The original citation (citation 29) for the Bayesian colocalization analysis method has been updated to Wallace (2021), which are more appropriate as methodological references (now citations 34).

5)、Section 2.6, lines 156-157 (posterior probability threshold): I have clarified that the posterior probability threshold of 0.8 is mentioned in the results section of citation 35. The reference (citation 20 and 35) has been updated and more detailed explanation has been added.

6)、Section 2.8, p.6, lines 171-172 (LD score regression): The original citation for LD score regression (citation 32) has been replaced with the correct original source, Bulik-Sullivan et al. (2015) (now citation 37).

7)、Comprehensive reference review: As per your suggestion, I have conducted a comprehensive review of the references throughout the manuscript, corrected any inappropriate citations, and added any missing references. I believe the revised manuscript now accurately reflects the sources used.

Thank you for your valuable comments, which have helped improve the rigor and clarity of the manuscript..

9. A few questions about the writing:

- p.5, line 146, the title of Section 2.6 “Bayesian co localization analysis” should be revised to “Bayesian colocalization analysis”.
- p.5, line 156, “We set the posterior probability threshold to greater than 0.8” should be revised to “We set the posterior probability threshold to be greater than 0.8”.
- p.6, line 167, “‘as a new endogenous exposure” should be revised to “as new endogenous exposures”.
- p.8, line 211, the title of Section 3.3 “Sensitivity analysis of the causal relationships between proteins and five OA” should be revised to “Sensitivity analysis of the causal relationships between proteins and five OA phenotypes”.
- I would suggest carefully reviewing the writing throughout the manuscript.

Response: Thank you for your valuable comments. We have carefully reviewed the manuscript and made the necessary revisions as per your suggestions:

1)、The title of Section 2.6 has been revised from "Bayesian co localization analysis" to "Bayesian colocalization analysis" to correct the spelling error.（line 138）

2)、The phrase "We set the posterior probability threshold to greater than 0.8" has been revised to "We set the posterior probability threshold to be greater than 0.8" to ensure grammatical correctness.（line 148）

3)、The phrase "'as a new endogenous exposure" has been revised to "as new endogenous exposures" to maintain consistency in the plural form.（line 159）

4)、The title of Section 3.3 has been revised from "Sensitivity analysis of the causal relationships between proteins and five OA" to "Sensitivity analysis of the causal relationships between proteins and five OA phenotypes" to provide more clarity.（line 208-209）

5)、We have carefully reviewed the writing throughout the manuscript and have made necessary corrections to improve the clarity and accuracy of the text.

We appreciate your thorough review and believe these revisions have improved the manuscript.

We sincerely thank the editors for our appreciation and evaluation of this paper, and we sincerely appreciate the reviewers for guiding us to improve the quality of the manuscript!

Please don’t hesitate to contact me if you have any questions.

Best wishes,

Xiaochun Wei，and Xueqin Rong
